# The Research Scholarly Output of Africa in Dermatology From 2012 to 2021: Focus on the Top 10 Dermatology Journals

**DOI:** 10.2196/41703

**Published:** 2023-03-15

**Authors:** Waseem Hassan, Saddam Hussain, Joao B T da Rocha

**Affiliations:** 1 Institute of Chemical Sciences University of Peshawar Peshawar Pakistan; 2 Departamento de Bioquímica e BiologiaMolecular Universidade Federal de Santa Maria Santa Maria Brazil

**Keywords:** Africa, dermatology, Scopus, bibliometry, bibliometric, scholarly research, research output, publish, academic journal, scientific research, scholarly journal, scientometric

Africa’s contributions to dermatology research have been underreported in the literature, prompting our investigation of the number and quality of scholarly output across the continent’s 49 countries. Using Scopus/SciVal, we analyzed publications from 2012 to 2021 and found only 4579 articles with 36,691 citations, indicating limited productivity. A total of 1804 (39.6%) papers, with 23,414 citations, were published with international collaboration.

To evaluate productivity by country, we used four indicators: number of publications, citations, citations per publication, and field-weighted citations impact. Egypt published the most documents (n=1688), followed by South Africa (n=685), Tunisia (n=388), Ethiopia (n=351), Morocco (n=290), Nigeria (n=249), and Kenya (n=206). The countries with the highest citations were Egypt (n=13,667), South Africa (n=8558), Morocco (n=2413), Kenya (n=2197), and Ethiopia (n=2176). Table 1 presents data for all 49 countries.

Journal ranking and metrics can indicate research quality, and Scopus categorizes journals into seven groups or quartiles. Of the 4579 African publications, 4267 are in one of the seven quartiles (Q1-Q7). Only 24 (0.56%) and 195 (4.01%) were published in the top 1% (Q1) and top 5% (Q2) of Scopus sources, respectively. The highest number of documents were in Q5 and Q6.

We also analyzed African contributions to the top 10 dermatology journals globally (Table 2). From 2012 to 2021, these journals collectively published 108,577 articles, but only 1060 (0.98%) came from Africa, with only 576 published without collaboration with high-income countries. The lack of investment, resources, and infrastructure in Africa likely contributes to low productivity, as well as the challenges faced by researchers in pursuing scientific careers in Africa [1].

Research is crucial for development and productivity growth, but Africa lags behind in investment. In 2011, while worldwide expenditure on research was 1.77% of the total global gross domestic product, Kenya spent only 0.1% and South Africa spent 0.76% of their gross domestic product on research [2,3]. This decline in research quality is attributed to insufficient spending. Only 2% of the 3000 publications from low-income countries are listed in MEDLINE, and only 10% of medical research is conducted in low-income nations. Even in the case of Ebola research, most of it was done in the United States [4].

African scholars must remain dedicated to addressing their continent’s problems and should consider stepping outside their comfort zones to pursue knowledge, develop long-term partnerships with high-income countries, and use applied research to bring new information to the continent [2,3]. Ongoing discussions among stakeholders, including local governments and research institutions, are essential for putting local research into practice. Regular engagement with regional and international researchers and policy makers is necessary to understand global concerns and priorities. To support these efforts, financial aid, research budgets, collaboration, and exchange programs are urgently needed.

**Table 1:** The scholarly output for all 49 countries.

Country/region	Scholarly output, n	Citations, n	Citations per publication	Field-weighted citation impact
Egypt	1688	13,667	8.1	0.96
South Africa	685	8558	12.5	1.21
Tunisia	388	2118	5.5	0.68
Ethiopia	351	2176	6.2	0.51
Morocco	290	2413	8.3	0.69
Nigeria	249	1788	7.2	0.6
Kenya	206	2197	10.7	0.87
Uganda	152	1672	11	0.85
Tanzania	124	1213	9.8	0.74
Malawi	65	571	8.8	0.75
Cameroon	60	462	7.7	0.54
Ghana	54	346	6.4	0.56
Botswana	53	277	5.2	0.46
Côte d'Ivoire	53	253	4.8	0.5
Zimbabwe	50	638	12.8	0.93
Burkina Faso	42	202	4.8	0.54
Senegal	41	316	7.7	0.79
Togo	36	141	3.9	0.43
Zambia	32	302	9.4	0.65
Benin	31	169	5.5	0.52
Rwanda	30	305	10.2	0.84
Sudan	30	351	11.7	1.1
Algeria	26	223	8.6	1.21
Libyan Arab Jamahiriya	21	791	37.7	1.88
Mali	19	117	6.2	0.57
Mozambique	17	149	8.8	0.89
Madagascar	12	43	3.6	0.3
Congo	10	51	5.1	0.39
Guinea	10	69	6.9	0.68
Democratic Republic Congo	9	38	4.2	0.61
Gabon	8	83	10.4	0.77
Mauritius	8	51	6.4	1.11
Reunion	8	50	6.3	1.03
Lesotho	7	23	3.3	0.32
Namibia	7	81	11.6	0.61
Liberia	6	7	1.2	0.09
Angola	5	13	2.6	0.54
Somalia	4	9	2.3	0.4
Central African Republic	3	41	13.7	1.16
Niger	3	13	4.3	0.42
Guinea-Bissau	3	37	12.3	1.12
Sierra Leone	3	15	5	0.45
Swaziland	3	23	7.7	0.71
Burundi	2	10	5	0.33
Gambia	2	27	13.5	1.19
Mauritania	2	14	7	0.99
Chad	1	3	3	0.44
Comoros	1	8	8	0.54
South Sudan	1	10	10	0.93

**Table 2:** The list of the top 10 journals with the total number of publications, number of countries involved, number of African countries, African total publications with collaboration, African total publications without collaboration, the top six African countries, and their contribution to each journal.

Title	Publications, n	Countries, n	African countries, n	Total African publications with collaboration, n	Total African publications without collaboration, n	Egypt, n	South Africa, n	Tunisia, n	Malta, n	Nigeria, n	Morocco, n
Journal of the American Academy of Dermatology	26,474	116	29	154	68	60	33	9	5	7	6
JAMA Dermatology	3638	83	17	18	2	1	3	2	1	3	0
American Journal of Clinical Dermatology	1452	62	6	14	8	5	4	2	1	1	0
Journal of the European Academy of Dermatology and Venereology	11,571	134	38	237	127	86	16	45	36	8	10
Experimental Dermatology	4480	74	8	36	10	18	8	5	0	0	1
Journal of Dermatological Science	3721	73	7	23	9	11	6	0	0	1	2
Clinics in Dermatology	3534	80	18	54	24	13	13	4	4	0	0
Journal of Investigative Dermatology	21,065	95	14	57	6	11	16	10	0	0	5
British Journal of Dermatology	29,828	123	35	439	306	66	229	20	19	22	0
Dermatologic Clinics	2814	61	8	28	16	4	17	0	1	0	0
